# Expression of *meis* and *hoxa11* in dipnoan and teleost fins provides new insights into the evolution of vertebrate appendages

**DOI:** 10.1186/s13227-018-0099-9

**Published:** 2018-04-27

**Authors:** Fernanda Langellotto, Maria Fiorentino, Elena De Felice, Luigi Caputi, Valeria Nittoli, Jean M. P. Joss, Paolo Sordino

**Affiliations:** 1 Dragonfly Therapeutics, Inc., 35 Gatehouse Drive, Waltham, MA 02451 USA; 2 Department of Pediatrics, Massachusetts General Hospital, Harvard Medical School, East Building 114, 16th Street, Charlestown, MA USA; 3 School of Biosciences and Veterinary Medicine, University of Camerino, Via R. Fidanza 15, 62024 Matelica, Italy; 4 Department of Integrated Marine Ecology, Stazione Zoologica Anton Dohrn, Villa Comunale, 80121 Naples, Italy; 5 Department of Biology and Evolution of Marine Organisms, Stazione Zoologica Anton Dohrn, Villa Comunale, 80121 Naples, Italy; 6 Biological Sciences, Macquarie University, Balaclava Road, North Ryde, Sydney, NSW 2109 Australia

**Keywords:** Fin-to-limb transition, *Neoceratodus*, Zebrafish, *Hoxa11*, *Meis*, Gene expression

## Abstract

**Background:**

The concerted activity of *Meis* and *Hoxa11* transcription factors is essential for the subdivision of tetrapod limbs into proximo-distal (PD) domains; however, little is know about the evolution of this patterning mechanism. Here, we aim to study the expression of *meis* and *hoxa11* orthologues in the median and paired rayed fins of zebrafish and in the lobed fins of the Australian lungfish.

**Results:**

First, a late phase of expression of *meis1.1* and *hoxa11b* in zebrafish dorsal and anal fins relates with segmentation of endochondral elements in proximal and distal radials. Second, our zebrafish in situ hybridization results reveal spatial and temporal changes between pectoral and pelvic fins. Third, in situ analysis of *meis1*, *meis3* and *hoxa11* genes in *Neoceratodus* pectoral fins identifies decoupled domains of expression along the PD axis.

**Conclusions:**

Our data raise the possibility that the origin of stylopod and zeugopod lies much deeper in gnathostome evolution and that variation in *meis* and *hoxa11* expression has played a substantial role in the transformation of appendage anatomy. Moreover, these observations provide evidence that the *Meis*/*Hoxa11* profile considered a hallmark of stylopod/zeugopod patterning is present in *Neoceratodus*.

**Electronic supplementary material:**

The online version of this article (10.1186/s13227-018-0099-9) contains supplementary material, which is available to authorized users.

## Background

Early development of both fore and hindlimbs of tetrapods involves condensation of endochondral skeleton within a mesenchymal lateral plate projection. In a proximo-distal (PD) sequence, first to form is the single element upper arm (stylopod), which articulates with the pectoral girdle. The second is the dual-element forearm (zeugopod) followed by the final region of the hand (autopod). This last region is the most variable, contributing to give rise to such limb specializations as wings and flippers. In the study of the evolution of tetrapod appendages, the majority of interest has been directed at the origin and variation of genetic mechanisms required for the formation of the autopod (hand, foot) [1–12]. Currently, there has been less interest in the evolution of the molecular underpinnings that control the clear division between the stylopod (humerus, femur) and zeugopod (ulna/radius, tibia/fibula) [13]. In particular, little is known about exactly when the changes in genetic processes occurred that were responsible for the endoskeletal patterns changing from a lobed fin pattern to that of a tetrapod limb.

Skeletal and molecular patterns support the hypothesis that the first fins may have originated as median fins in stem group gnathostomes and that paired fins are unique to jawed vertebrates and their pre-jawed relatives [14, 15]. Primitively, endoskeletal patterns of median fins consisted of morphologically identical and unsegmented radials (early craniates, e.g., *Myllokunmingia*), which later became segmented in two (basal gnathostomes) or three sections (acanthopterygians) [16]. The first paired fins for which there is evidence of endoskeletal support appeared in a lineage of jawless fishes that led to the gnathostome stem group. The pectoral fins of ancestral fishes possessed an endoskeleton consisting of proximal radials (basals) articulating directly with their girdles [17–19]. In living fishes, the number of radials is varied. The usual pattern in elasmobranchs is tribasal, made up of a propterygium, mesopterygium, and metapterygium [20], while the numbers of basals are much more diverse in teleost fishes and other members of the ray-finned clade (for an overview of actinopterygian paired fin anatomy, see [21]). Within actinopterygians, teleost fishes have lost the most posterior radial, the metapterygium [14]. Paleontological data suggest that the tetrapod limb originated from sarcopterygian fins by transformation of the endochondral architecture and loss of distal dermoskeleton [1–3]. Living sarcopterygians have retained exclusively the metapterygial elements and have lost the propterygium and mesopterygium [22]. In gnathostome pectoral fins, proximal radials articulate with distal row of smaller radials that originate by segmentation or de novo condensation [19]. The second set of paired appendages, the pelvic fins, appeared later in jawed vertebrates and are generally simpler in pattern, with an endoskeleton consisting of few rod-like basals [23–26]. A reductive trend of the number of radials resulting in the loss of basals is observed in pelvic fins of actinopterygians [22, 27].

Genetic approaches have led us to a point where the developmental mechanisms responsible for stylopod and zeugopod formation have been identified in limb. This process arises from the combined action of two genes encoding master regulators of transcription that have been identified as determinants of proximal and distal limb elements. The expression of *Meis* genes specifies stylopod cell identities, possibly in response to diffusible retinoic acid from flank cells, while *Hoxa11* genes are critical for normal growth rates and differentiation in the zeugopod under the influence of FGF signaling from the apical ectodermal ridge (AER) [28–33]. Spatial and temporal signatures for *Meis* and *Hoxa11* clearly distinguish between the stylopod and the zeugopod subdivisions and can be used as developmental landmarks for PD regionalization.

There is paucity of information about how the stylopod-zeugopod developmental program has changed during evolution and how this relates to morphological diversification. Chondrichthyans (sharks, rays, chimeras) retain most plesiomorphic features of primitive gnathostomes, including both midline and paired fins, and are a good model for phylogenetic sampling. Expression data are available pertaining paired fins of the small-spotted catshark, *Scyliorhinus canicula* [34–37] and the little skate, *Leucoraja erinacea* [38], making cartilaginous fishes a suitable outgroup to the actinopterygian fishes + sarcopterygians (lungfish, coelacanths and tetrapods) for testing hypotheses concerning the evolution of PD patterning mechanisms [39]. In chondrichthyans, pectoral fins develop by condensation of undifferentiated mesenchyme and by apoptosis [34]. During this process, *meis* and *hoxa11* are expressed proximally and distally, respectively, suggesting that some aspects of the genetic program for limb PD patterning are shared among gnathostomes [35]. Yet, evidence of a physical boundary between expression patterns, as it is observed in limbs, is missing [34–38]. The ray-finned zebrafish and paddlefish are independently derived actinopterygian taxa with a duplicated genome (Whole Genome Duplication, WGD) [40]. In the pectoral fins of these fishes, *hoxa11* and *meis* expression domains do not form a PD boundary and, in addition, gene expression wanes and ceases before radial formation [35–37, 41–45]. However, these data should be interpreted with caution when considering that zebrafish is a highly derived teleost and paddlefish is a living representative of chondrosteans and, as such, sister group to the neopterygians. The HoxA cluster generated by WGD appears to be inactive, with only a single paralog of each 5′ gene expressed in the developing fin [36, 46, 47], making necessary to be aware of which possible *hoxa11* paralogue (a or b) is examined. To our knowledge, there is no reported evidence of *hoxa11* and *meis* gene expression patterns during the development of paired fins of sarcopterygian fishes and in median or pelvic rayed fins of actinopterygian fishes.

While sarcopterygians primitively had polybasal paired fins [48], crown taxa appear to possess a one to two, proximo-distal ratio of appendicular cartilages or bones that are morphologically homologous to the stylopod and zeugopod of tetrapod limbs [49–53]. To date, molecular markers have not been applied to back up this morphological identification. This is primarily because only a handful of lungfish and coelacanth species have survived as sarcopterygian fish alive today. Since all tetrapods are also sarcopterygians, and Dipnoi as a whole are generally supported as the sister group of Tetrapoda, there is really only one fish whose paired fins are worth, by far, testing molecular markers for stylopod and zeugopod development. Coelacanths (only 2 living species) are deep-sea marine viviparous species both of which are listed as endangered and are less closely related to early tetrapods [54, 55]. Living lungfish occur in 2 families, the Lepidosirenidae (5 species) and the Ceratodidae (single species, *Neoceratodus forsteri*). It is only this latter that occurs close enough to habitation to make it feasible for molecular developmental study to compare with tetrapod and ray-finned fish paired fin early development to be carried out [56–59]. The lobed fins of *Neoceratodus* have an elongated axis generally considered as metapterygial, with pre- and post-axial radials [14]. A closer analysis of the morphological and genetic pathways in the development of paired fins in *Neoceratodus* may have implications for the developmental changes involved in the evolution of a paired lobed fin into a tetrapod limb.

Experiments on fish at other phylogenetic positions are needed to draw homologies based on comparative gene expression. Here, we aim to track the expression of *Meis* and *Hoxa11* genes in median and pelvic fins of zebrafish, and in paired fins of the Australian lungfish. A comparative analysis may give clue to the degree of variation in the activity of these developmental landmarks. How flexible PD patterning mechanisms are in jawed vertebrate appendages is still a poorly explored issue, nor is the sequence and timing of this process clear.

## Methods

### Embryos

Zebrafish embryos were obtained from natural spawning of wild-type (AB*) fish. Lungfish were obtained as fertilized eggs from dedicated spawning ponds established at Macquarie University and reared to appropriate stages prior to fixation in buffered 4% paraformaldehyde. Lungfish were fixed for 24–48 h (depending on size), rinsed in buffer, transferred to 100% methanol, and stored at − 20 °C.

### Cloning

*Neoceratodus forsteri* RNA was extracted from stage 40–42 embryos (RNeasy, Qiagen), followed by cDNA synthesis (Smart Race, Clontech). To amplify *meis* cDNAs, full-length nucleotide and amino acid sequences of vertebrate homologs were aligned to design degenerate oligonucleotides in conserved regions: *Meis*-*fw* GCTGGCHCTSATYTTYGARAARGYGA and *Meis*-*rev* CGTCKTCKKGCRTTRATRAACCARTT. PCR fragments of lungfish *meis1* and *meis3* genes were cloned, sequenced and analyzed. *Neoceratodus hoxa11* full-length cDNA was kindly provided by M. Sutija who amplified it with *Hoxa11*–*fw2* TCCDGATTTCTCCAGCCTSCC and *Hoxa11*–*rev1* CAGATTTTAACTTGACGGTCGGT based on previous sequence [60].

### Phylogeny

Amino acid sequences of lungfish Hox and *Meis* and of their vertebrate orthologues were gathered from public databases and aligned using Muscle v3.8.31 [61]. The alignments were visually checked using Bioedit v7.0.5.3 [62]. *Hoxa11* affiliation was confirmed with the program HoxPred (URL: http://cege.vub.ac.be/hoxpred) [63]. The best fitting amino acid substitution models for the Hox and *Meis* alignments were selected using ProtTest 3 [64], using the “fast” option and under a full coverage of the amino acidic substitution matrices and of the specific corrections therein proposed. The Akaike information criterion (AIC), the Akaike second-order information criterion (AICc) and the Bayesian information criterion (BIC) selected the JTT + I + G as the best fitting model for the Hox (γ = 2.078) and *Meis* (γ = 0.572) alignments. The ProtTest 3 results were used to constrain “Neighbor Joining” (NJ) and “Maximum Likelihood” phylogenies. The NJ constrained phylogeny was inferred using MEGA5 [65] under the JTT model of amino acidic evolution and performing 10,000 bootstrap replicates. The ML phylogeny was inferred using the software PhyML 3.0 [66], performing 1000 bootstrap replicates. Finally, Maximum Parsimony (MP) phylogeny was inferred after performing 500 bootstrap replicates using the close neighbor interchange (CNI) on random tree search method and 1000 initial trees on MEGA5. The topologies inferred with the three methods are substantially similar for both *Meis* and Hox protein phylogenetic analyses. The nucleotide sequences of lungfish *meis1*, *meis3* and *hoxa11* genes are deposited in GenBank database under the accession numbers: [GenBank sequence submission ongoing].

### Labeling

For zebrafish (*n* = 10 embryos per experiment; data gathered from 5 embryos), standard methods for whole-mount in situ hybridization (WISH) with digoxigenin-labeled riboprobe and for single color labeling were used. For lungfish (*n* = 4–5; 2–3), single WISH with digoxigenin-labeled riboprobes were carried out according to standard methods modified for better riboprobe penetration by elongating the proteinase K treatment step [56]. No staining was detected when using sense probes. The precise expression boundary between *Neoceratodus meis* and *hoxa11* genes could not be adequately resolved since the *hoxa11* riboprobe did not work in double WISH experiments. To correlate gene expression patterns with cartilage condensations, Alcian Blue staining was performed before WISH. Labeled fins were removed with Dumont forceps and mounted in glycerol for imaging with the AxioImager M1 (Zeiss) microscope or processed for cross sections in Epoxy resin. Differential Interference Contrast microscopy images were acquired with a Zeiss Axio Imager M1 microscope equipped with an Axiocam digital camera. Figure plates were made with Adobe Photoshop CS4. Brightness/contrast and color balance adjustments where applied, were applied to the entirety of the image and not to parts thereof.

## Results

### *Meis1.1* and *Hoxa11b* expression during cartilage segmentation in dorsal and anal fins of zebrafish

**Fig. 1:**
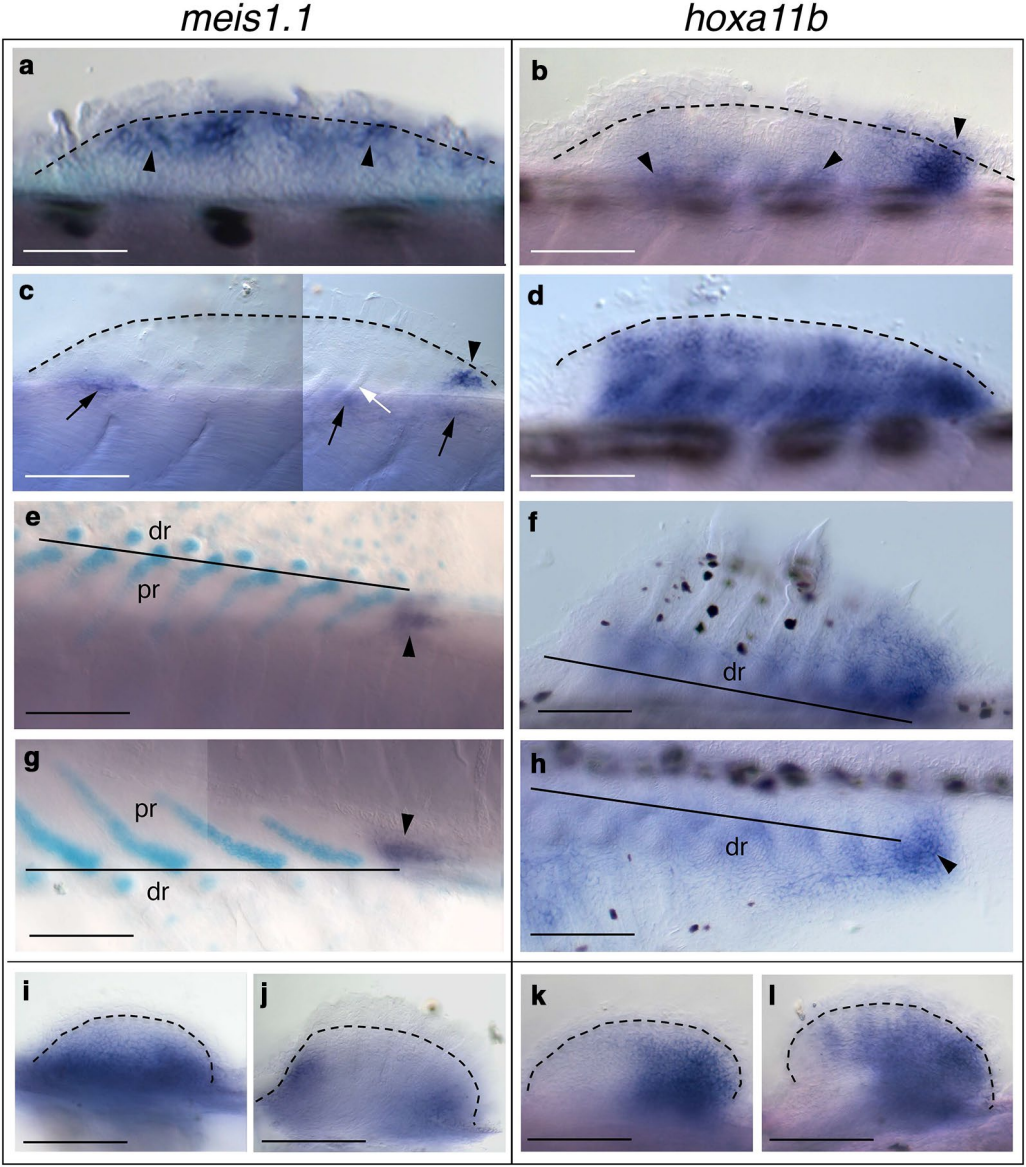
*meis1.1* and *hoxa11b* expression in zebrafish fins. **a**–**f** Dorsal, **g**, **h** anal and **i**–**l** pelvic fins. **a**–**d**, **f**, **h**, **i**–**l** WISH and **e**, **g** Alcian Blue staining followed by WISH. **a**–**c**, **e**, **g**, **h** Arrowheads indicate fin mesenchyme cells expressing *meis1.1* and *hoxa11b*. **c** The white arrow indicates unsegmented radial; **c** the black arrows indicate WISH staining in dorsal somite cells. **a**–**d**, **j**, **l** The dashed lines indicate distal limit of endochondrogenic mesoderm below the finfold in **a**–**d** dorsal and **j**, **l** pelvic fins. **e**–**h** The solid lines indicate the boundary between proximal and distal radials in **e**, **f** dorsal and **g**, **h** anal fins. **a**–**f**, **i**–**l** Anterior to left, distal to top; **g**, **h** anterior to left, distal to bottom. Fish length is **a**–**c** 3.6–4.4 mm, **d** 4.0–5.2 mm, **e**–**h** 5.6–6.1 mm, **i**, **k** 4.1–5.3 mm, **j**, **l** 5.0–6.4 mm. *Abbreviations*: dr, distal radial; pr, proximal radial. Scale bars are 100 μm (**a**, **b**, **f**, **g**), 50 μm (**c**–**e**, **h**–**l**), and 70 μm (**k**–**n**)

We investigated *meis* and *hoxa11* gene expression with respect to chondrogenesis in dorsal and anal fins of zebrafish. By whole-mount in situ hybridization (WISH) experiments, *meis3.1* and *hoxa11a* were not expressed during the development of these median fins. In contrast, *meis1.1* and *hoxa11b* genes showed three distinct phases of expression. When the dorsal fin bud is hardly visible, flattened stacks of chondrocytes form undivided cartilage rods (notochord length, NL = 3.6–4.4 mm; somites 15th to 17th). At this stage, *meis1.1* was first expressed in a narrow territory immediately under the apical ectodermal ridge (AER) (Fig. 1a). On the other hand, cells positive for *hoxa11b* expression were seen at the base of the fin bud and also in the posterior fin mesenchyme (Fig. 1b). Before radial segmentation (NL = 4.0–5.2 mm), *meis1.1* expression had become posterior; *meis1.1* transcript was observed also in dorsal somite cells (Fig. 1c). With the onset of radial segmentation (NL = 5.0–5.4 mm), *hoxa11b* expression extended across the fin except the anterior margin, in a way reminiscent of late-phase expression of *hoxa11* orthologous in paired rayed fins and limbs [38–40, 43] (Fig. 1d). At late stage of radial segmentation (NL = 5.6–6.1 mm) during the posterior expansion of the radials array, *meis1.1* mRNA signal was seen at the site of chondrogenesis of the last proximal radial (Fig. 1e, g), and *hoxa11a* expression in the mesenchyme between distal radials (Fig. 1f, h). Thus, late-phase expression of *meis1.1* and *hoxa11b* correlates with endoskeletal regionalization along the PD axis.

### Expression of *Meis1.1* and *Hoxa11b* in pelvic fins of zebrafish

Studies of *meis1* and *hoxa11* gene expression patterns in pectoral fins of shark, paddlefish and zebrafish revealed some substantial differences [36, 37, 41–45]. Here, we are interested to investigate whether a similar phylogenetic variation exists also within lineages, *i.e.,* between anterior and posterior paired appendages of teleosts. The first phase of *meis1.1* and *hoxa11b* expression in zebrafish pelvic fin buds was very similar to pectorals, with proximal and posterior mRNA restriction, respectively (NL = 4.1–5.3 mm) (Fig. 1i, k) [41–43]. Later, both transcription factors were transcribed during chondrification in pelvics (NL = 5.0–6.4 mm). In particular, transcriptional activity of *meis1.1* was restricted proximally to anterior and posterior margins (Fig. 1j), and *hoxa11b* expression was anteriorly expanded, resembling the second phase of *hoxa11* regulation in pectoral fins and limbs [45, 67] (Fig. 1l). Late-phase expression of *meis1.1* in pelvic fins is particularly interesting because in pectoral fins this signal is rapidly polarized to the proximal terminus during fin skeleton reorganization (Additional file 1).

### Evidence of a molecular stylopod/zeugopod boundary in paired fins of *Neoceratodus*

To discover whether the development of paired lobed fins displays mRNA domains that are consistent with the stylopod/zeugopod homology supported by morphological studies, we examined *meis1*, *meis3* and *hoxa11* transcriptional profiles in lungfish, under the assumption that genes involved in PD patterning should behave similarly in paired lobed fins and limbs. PCR fragments of *N. forsteri* genes were obtained using degenerate primers designed against conserved regions of such genes in other species on total cDNA from stage 40–48 whole embryos. The affiliation of *Neoceratodus meis1* (718 bp), *meis3* (732 bp) and *hoxa11* (774 bp) cDNA sequences was suggested in a Blastx search against NCBI non-redundant protein sequences (nr) and was clearly indicated by sequence alignments (Additional file 2: Fig. 2a, b). The position of *N. forsteri* predicted proteins within the topologies is consistent with their basal location to tetrapods [54, 55] (Additional file 2: Fig. 2c, d).

**Fig. 2:**
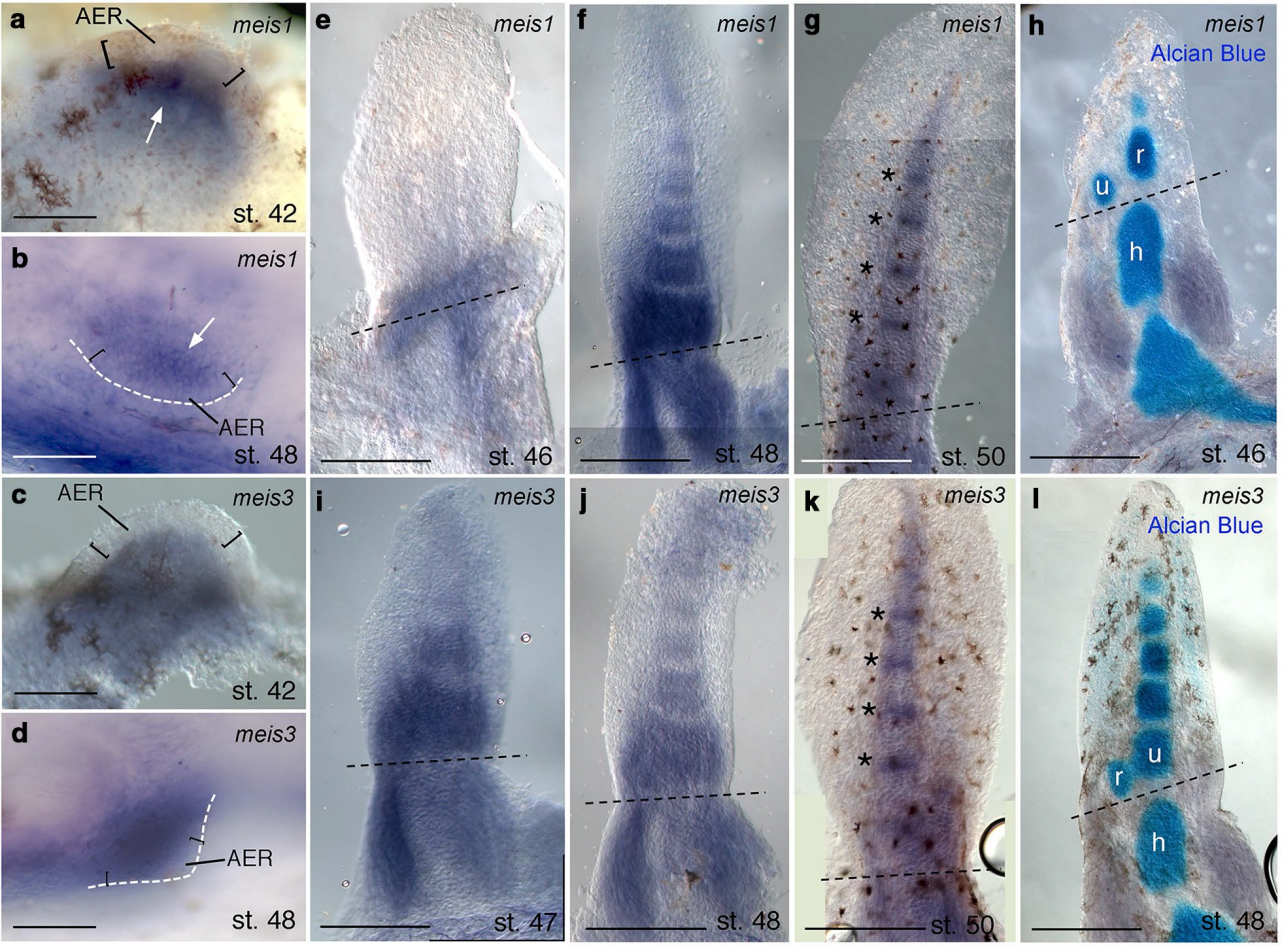
*meis1* and *meis3* expression in *Neoceratodus* lobed fins. Whole-mount in situ hybridization in **a**, **c**, **e**–**l** pectoral and **b**, **d** pelvic lobed fins. **a**, **b** White arrowheads indicate *meis1* transcript under the AER in **a** pectoral and **b** pelvic fin buds. **c**–**g**, **i**–**k** *meis3* expression in the whole bud of **c** pectoral and **d** pelvic fins. **b**, **d** The white dashed lines highlight the margin of paired pelvic fin buds. **a**–**d** Square brackets indicate the AER. The black dashed lines indicate the stylopod-zeugopod boundary at chondrogenesis stages. **e**–**g**, **i**–**k** From st. 46 to st. 50, **e**, **i** *meis1* and *meis3* transcripts are first localized in the proximal cells of the pectoral lobed fin, **f**, **j** after they extend gradually in a PD striped pattern around cartilage condensations, **g**, **k** and then they show intersegmental expression (asterisks). **h**, **l** Double Alcian Blue/WISH labelings show proximal expression across the humerus region and the first transversal stripe near radius/ulna cartilages. *Abbreviations*: AER, apical ectodermal ridge; c, cartilage; h, humerus; m, mesenchyme; r, radius; u, ulna. Anterior to left; distal to top. Scale bars are 100 µm (**a**–**d**), 80 µm (**e**, **f**, **i**) and 50 µm (**g**, **h**, **j**–**l**)

WISH was performed by use of digoxigenin-labeled antisense riboprobes synthetized from the entire cDNA fragments. The *hoxa11* probe spans the entire coding sequence, while *meis1* and *meis3* probes target the entire *MEIS* domain and most of the homeodomain (Additional file 3). In the nascent paired fins of *Neoceratodus*, *meis1* expression is restricted in mesenchymal cells under the AER, while *meis3* mRNA signal is expanded in the entire bud mesenchyme (st. 42, pectorals; st. 48, pelvics) (Fig. 2a–d) (Additional file 4: Fig. 4a). During chondrogenesis (st. 46–48), *meis1* and *meis3* were diffusely expressed in the proximal third of the pectoral fin, and in a segmentally reiterated pattern extending distally during development (Fig. 2e, f, i, j) (Additional file 4: Fig. 4b, c). Later in development, *meis1* and *meis3* expression was present in the intersegmental mesenchyme (st. 50) (Fig. 2g, k). Combining WISH and Alcian Blue (st. 46–48), the proximal domain of *meis1* and *meis3* expression was recognized in the region where the humerus cartilage differentiates (Fig. 2h, l).

**Fig. 3:**
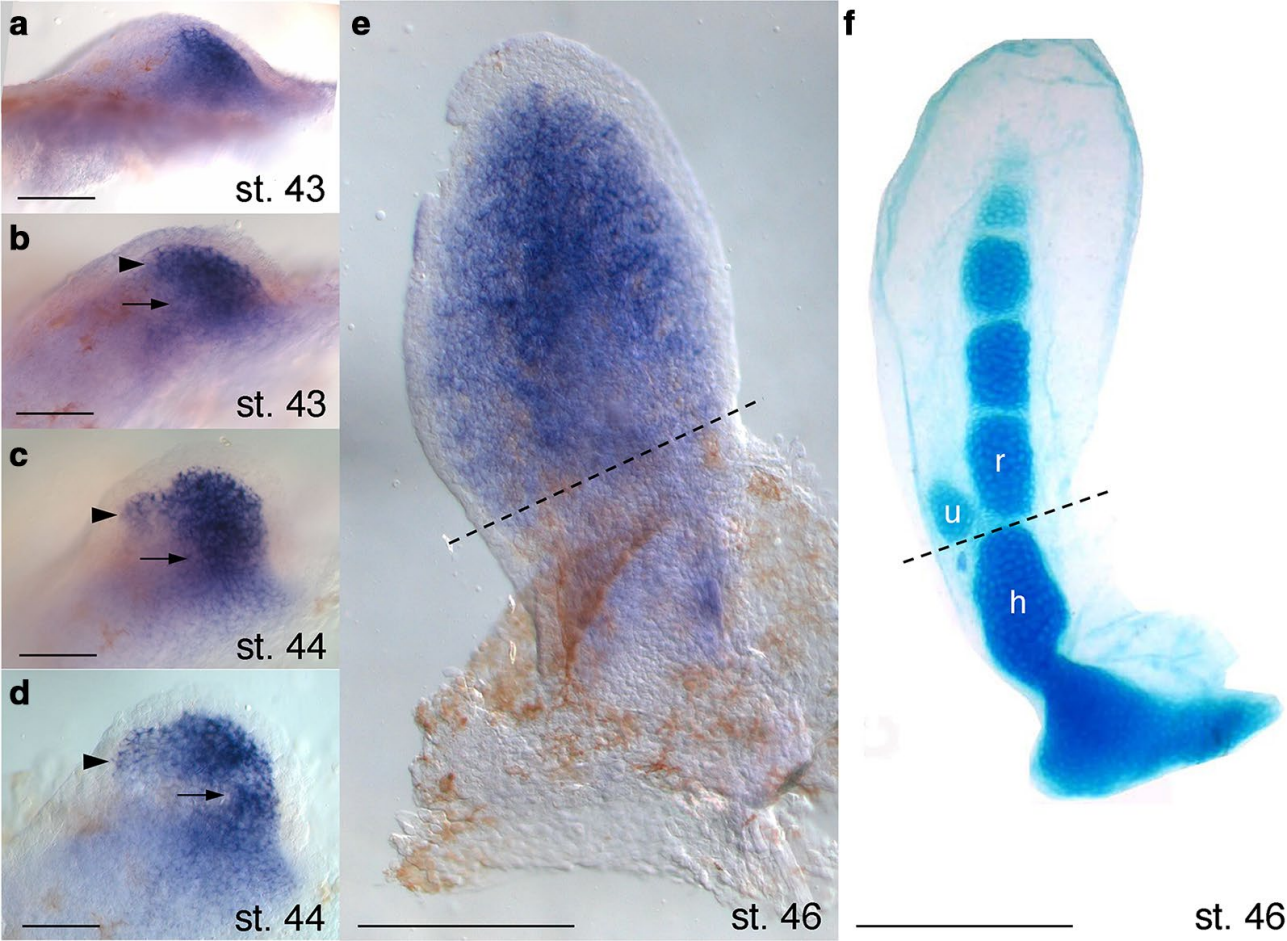
*hoxa11* expression in *Neoceratodus* lobed fins. WISH in pectoral lobed fins from early budding to early chondrogenesis. **a** Distribution of *hoxa11* transcript in the posterior bud. **b**–**d** The arrowheads indicate expression in anterior cells at the distal margin of the lungfish pectoral fin. **b**–**d** The arrows indicate a PD discontinuity of low, if any, expression in the early phase of *hoxa11* activity. **e**, **f** Comparison of single WISH and Alcian Blue staining indicates that transcription is depleted in those cells that will ultimately build the humerus (below dashed line). *Abbreviations*: h, humerus; r, radius; u, ulna. Distal to top, anterior to left. Scale bars are 100 µm (**a**–**d**) and 50 µm (**e**, **f**)

In the paired fin buds of the Australian lungfish, the expression of *hoxa11* was restricted to a posterior area of mesenchymal cells named the polarizing region, as seen in other tetrapod appendages (st. 43) (Fig. 3a) [67]. During fin outgrowth, *hoxa11* transcription was turned on in more anterior cells in the distal aspect of the fin; of note, a PD discontinuity in the unique and continuous domain became visible between st. 43 and 44 (st. 43–44) (Fig. 3b–d). Poor ability of *hoxa11* mRNA riboprobe to generate a detectable signal in combination with Alcian Blue did not allow relating gene expression to anatomy. However, comparable st. 46 embryos suggest that *hoxa11* expression is excluded proximally from the region where the humerus cartilage differentiates (Fig. 3e, f). *Neoceratodus hoxa11* was transcribed in several other developing tissues, including neural tube, somites and digestive system (Additional file 5).

## Discussion

**Fig. 4:**
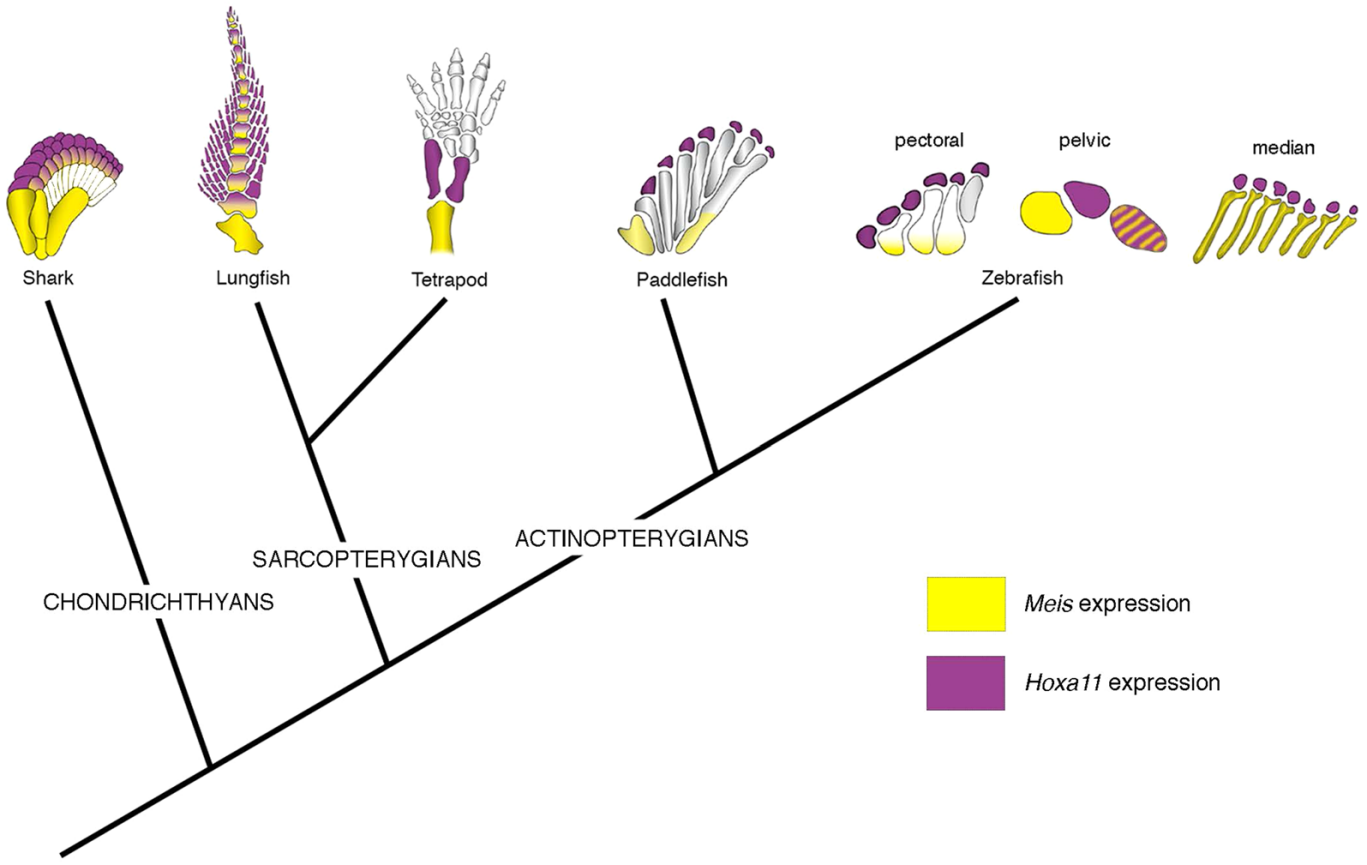
Late-phase *Meis* and *Hoxa11* expression in vertebrate appendages. The tree shows phylogenetic relationships of chondrichthyans, sarcopterygians and actinopterygians. *Meis* (yellow) and *Hoxa11* (purple) expression domains at late stage of fin/limb development superimposed on the appendicular skeleton for each taxon

Here, findings raise the possibility that some aspects of the role of *meis* and *hoxa11* in PD regionalization also operated in the development of median fins of early vertebrates. It was reported that median fins share several anatomical and molecular traits with limb development, indicating that some features of the primitive genetic mechanism for median fin development have been recruited in a new position to provide patterning information for paired pectoral fins [10, 15, 34]. This hypothesis is further supported by the correspondence in timing and pattern of expression of segmentation genes in median and paired fins [68, 69]. Here, we found that late-phase expression of *meis1.1* and *hoxa11b* is associated with the formation of proximal and distal radials, respectively, in the dorsal and anal fins of zebrafish, a condition that is neither found in paired fins of derived nor basal lineages of actinopterygian fishes [37, 41–45]. Late-phase restriction of *meis1.1* and *hoxa11b* expression patterns in dorsal and anal fins of zebrafish suggests that the evolutionary roots of limb subdivisions rely on a common theme in the development of vertebrate appendages. These domains of *meis1.1* and *hoxa11b* expression are not considered stylopod- and zeugopod-like, as they inform positional cues that have probably been important for generating morphological diversity in all animal appendages [70–72]. To confirm or falsify this hypothesis, expression of *meis* and *hoxa11* orthologues as well as of other regionalization and segmentation genes must be investigated in chondrichthyan (e.g., small-spotted catshark) and in non-teleostean actinopterygian clades (e.g., spotted gar, paddlefish and bichir) in detail. After the evaluation of catshark *hoxa11* and *meis1* patterns by Sakamoto and coauthors [35], and of paddlefish *hoxa11* and *meis2* patterns by Tulenko and coauthors [37], it became clear that a true PD boundary is not present in the paired fins of chondrichthyan and actinopterygian fishes. However, the presence of a late-phase PD pattern of *hoxa11b* and *meis1*.*1* in zebrafish dorsal and anal fins suggests that some aspects of the genetic program for limb PD patterning are shared among gnathostomes (Fig. 4),

Ray-finned fishes show a significant range of morphological and functional disparities in their pectoral and pelvic fins, and the reductive evolution of this dimorphism in tetrapod appendages is considered one of the most important differences between the two groups [19, 25, 26, 73]. Regulation of *meis1.1* and *hoxa11b* expression is different in zebrafish pectoral and pelvic fins [41, 42]. The domain of *meis1.1* expression in pelvic fins extended proximo-distally to the anterior and posterior mesenchyme, in support of the hypothesis of a proximalized skeleton phenotype (Fig. 1j). Differential expression of transcription factors between paired fin types of zebrafish may also reflect a developmental change in signaling pathways [74–77]. We hypothesize that spatial and temporal changes in the expression of these genes correlate with the complexity of fin skeletal patterns and with flexible morphology and function that have probably facilitated the adaptations of paired fins to various environments.

The stylopod/zeugopod (SZ) boundary of *meis* and *hoxa11* in the sarcopterygian lungfish pectoral fins provides a molecular basis for the phylogenetic homologies between tetrapod forelimb and lungfish pectoral fins [49, 50]. In our study, *meis1* and *hoxa11* gene expression domains in *Neoceratodus* paired pectoral fins closely resemble those of tetrapod limbs, in that both structures have a S/Z boundary during their growth and patterning. While all four clusters of tetrapod *Hox* genes are expressed, 5′ genes of the A cluster are most significant in specifying proximo-distal patterning and the D cluster in antero-posterior orientation [2, 78, 79]. *Hox* gene expression during the development of tetrapod limbs occurs in two distinct phases associated with different regions of the limb. During outgrowth of paired lobed fins, a unique extended domain of *hoxa11* expression becomes disconnected along the proximo-distal axis by the creation of a zone with cells producing low mRNA. The question of whether the region with low *hoxa11* expression observed in lungfish fins possesses a “mesopodial identity”, as a zone of transition between two distinct phases of *hoxa11* expression, or it is merely labeling the separation between elongated bones (the humerus and the ulna/radius), “*cannot be solved by the mere contemplation of expression patterns*” [78] and thus requires more evidence [44, 78, 80]. Here, the fundamental difference in *Hox* gene expression between a dipnoan fin and a tetrapod limb is the overlap between the expression domains of *hoxa11* and *hoxd13* throughout the region representing lobed fin radials [56, 81]. Co-expression of these two genes also occurs during rayed fin development, but it is very early in comparison with lungfish fins where it is found after the homologues of the stylopod and zeugopod have begun to condense [40, 43, 44]. Thus, in terms of *Hox* gene expression, the paired fins of *N. forsteri* may be viewed as lacking an autopod (*hoxd13* expression is not exclusive to a distal region beyond *hoxa11* expression).

## Conclusions

Our findings for *Neoceratodus* specifically highlight the homology of stylopod and zeugopod elements described previously from fossil and morphological evidence. This leaves only the autopod as a possible tetrapod invention. The reiterated *Meis* expression is a clear separation from the position in tetrapod limbs and must represent a distal developmental pattern of the paired lobed fin of the Australian lungfish. If we consider the segmental pattern of *Meis* gene expression in lobed fins and its absence in tetrapod limbs to be a clear fin *vs* limb patterning, it calls into question the concept of a metapterygial axis equating to various autopodal parts [82]. The evidence in paddlefish pectoral fins of proximal *meis2* expression makes us favor the hypothesis of distal *meis1* and *meis3* expression pattern being simply an independently derived dipnoan specialization. Certain apomorphies of paired lobed fins, such as distal *Meis* expression, are perhaps to be expected given the current phylogenetic position of dipnoans relative to tetrapods, as the extant sister group to the Tetrapoda, but more phylogenetically distant relative to a number of fossil sarcopterygians [83, 84]. In effect, proximo-distal sequence of *Meis* expression along the metapterygial axis of lobed fins seems to be supportive of the role of segmentation for limb development [85]. Morphologically, lungfish fins have both stylopod and zeugopod equivalents. One century after its proposal, we provide the first molecular validation of W. K. Gregory’s breakthrough view that the upper and forearm of the tetrapod limb originated from the skeletal elements of crown-sarcopterygian fishes [49, 50].

## Additional files

**Additional file 1: Fig. 1** *meis1.1* expression and chondrogenesis in zebrafish pectoral fins. mRNA labeling shows proximal restriction during cartilage remodeling.

**Additional file 2: Fig. 2** Alignment and phylogenetic analysis of *Neoceratodus* proteins. Partial protein-translated alignment and molecular phylogeny in ortholog identification of *Neoceratodus meis1*, *meis3* and *hoxa11.*

**Additional file 3: Fig. 3** Position of lungfish riboprobes. WISH riboprobes spanning mature mRNAs.

**Additional file 4: Fig. 4** *meis3* expression in developing pectoral fins of *Neoceratodus*. Transversal sections of in situ results at st. 42 and 47.

**Additional file 5: Fig. 5** *hoxa11* expression in lungfish. *In situ* results in nervous system, tail and digestive tract.
