# Supplementary material for: Expression of meis and hoxa11 in dipnoan and teleost fins provides new insights into the evolution of vertebrate appendages
Source: EvoDevo. 2018 Apr 27;9:11. doi: 10.1186/s13227-018-0099-9 (PMC5924435; doi:10.1186/s13227-018-0099-9)
Supplement: Supplementary file 1 — Additional file 1: Fig. 1 meis1.1 expression and chondrogenesis in zebrafish pectoral fins. mRNA labeling shows proximal restriction during cartilage remodeling. [file 13227_2018_99_MOESM1_ESM.pdf]

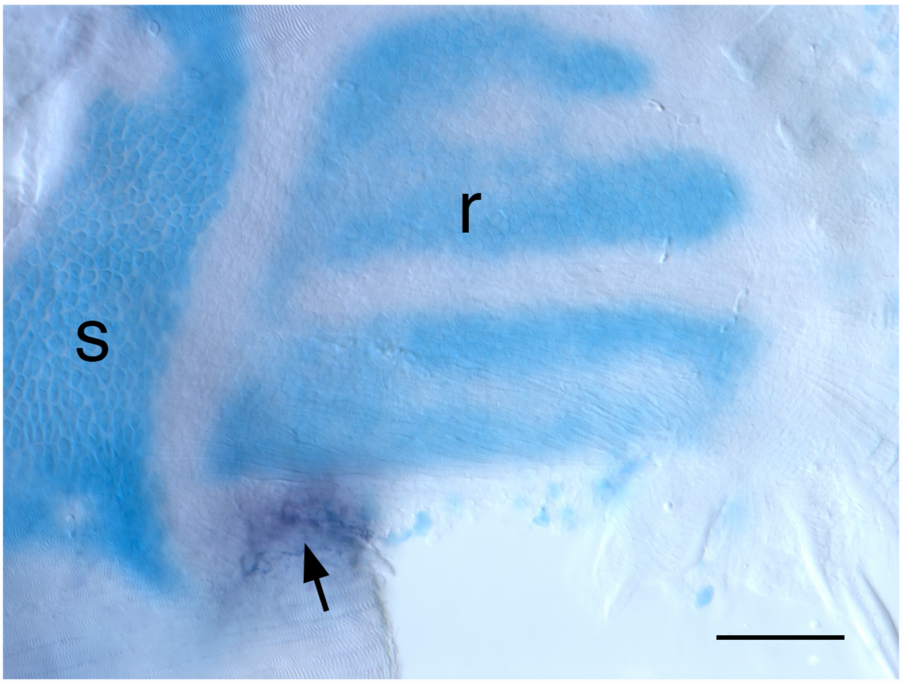

Additional file 1: Figure 1. *meis1.1* expression and chondrogenesis in zebrafish pectoral fin. Dorsal view of Alcian Blue staining followed by WISH. *meis1.1* transcript is restricted to proximal-posterior margin (arrow) during cartilage remodelling. Abbr.: s, scapulocoracoid; r, radial. Scale bar: 100 µm.
