# Supplementary material for: Expression of meis and hoxa11 in dipnoan and teleost fins provides new insights into the evolution of vertebrate appendages
Source: EvoDevo. 2018 Apr 27;9:11. doi: 10.1186/s13227-018-0099-9 (PMC5924435; doi:10.1186/s13227-018-0099-9)
Supplement: Supplementary file 2 — Additional file 2: Fig. 2 Alignment and phylogenetic analysis of Neoceratodus proteins. Partial protein-translated alignment and molecular phylogeny in ortholog identification of Neoceratodus meis1, meis3 and hoxa11. [file 13227_2018_99_MOESM2_ESM.pdf]

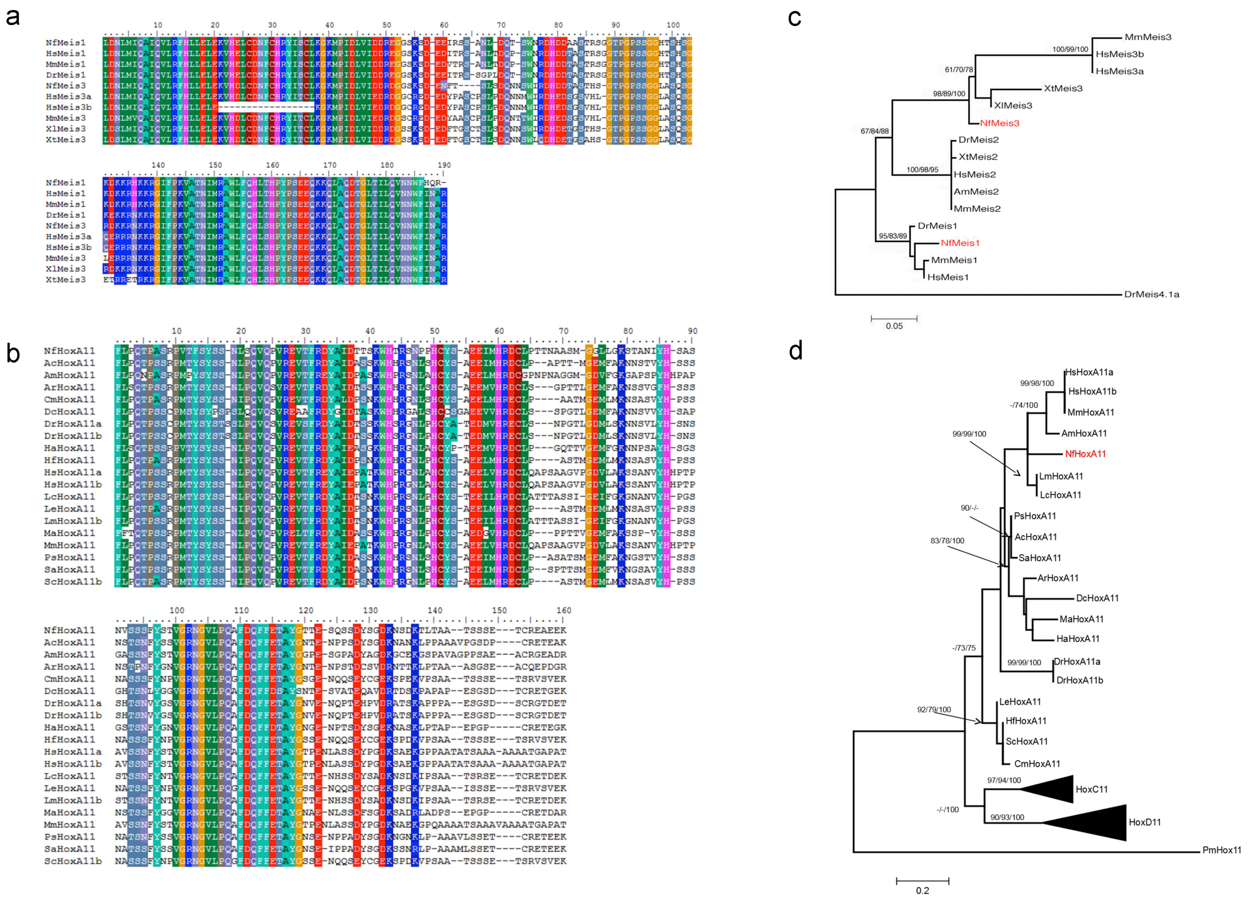

Additional file 2: Figure 2. Alignment and phylogenetic analysis of *Neoceratodus* proteins. (a,b) Partial protein-translated alignment of lungfish Meis1 (NfMeis1), Meis3 (NfMeis3) and Hoxa11 (NfHoxa11). (c,d) Phylogeny of *Neoceratodus* Meis1, Meis3 and Hoxa11 within higher vertebrate orthologues. Phylogeny of *Neoceratodus* Hoxa11 within Hox proteins of the vertebrate parologue groups 9-14. Support values at nodes represent bootstrap percentage for the Neighbour Joining (10000 bootstraps), Maximum Parsimony (1000 bs) and Maximum Likelihood (1000 bs) topologies. Abbreviations: Am, *Ambystoma mexicanum*; Cm, *Callorhinchus milii*; Dr, *Danio rerio*; Hf, *Heterodontus francisci*; Hs, *Homo sapiens*; Ib, *Ichthyophis bannanicus*; Le, *Leucoraja* erinacea; Lm, *Latimeria menadoensis*; Mm, *Mus musculus*; Nf, *Neoceratodus forsteri*; Pa, *Protopterus annectens*; Rt, *Rana temporaria*; Xl, *Xenopus laevis*; Xt, *Xenopus tropicalis*.
