# Supplementary material for: Expression of meis and hoxa11 in dipnoan and teleost fins provides new insights into the evolution of vertebrate appendages
Source: EvoDevo. 2018 Apr 27;9:11. doi: 10.1186/s13227-018-0099-9 (PMC5924435; doi:10.1186/s13227-018-0099-9)
Supplement: Supplementary file 3 — Additional file 3: Fig. 3 Position of lungfish riboprobes. WISH riboprobes spanning mature mRNAs. [file 13227_2018_99_MOESM3_ESM.pdf]

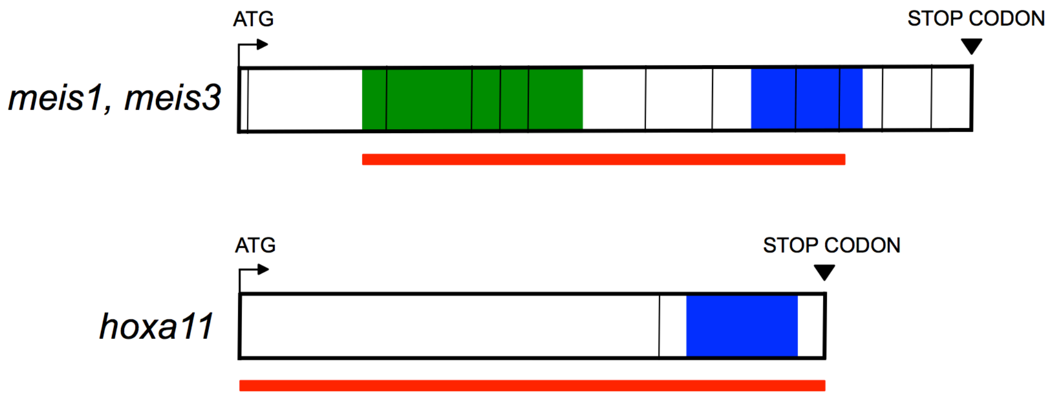

Additional file 3: Figure 3. Position of lungfish riboprobes. Schematic of *meis1*, *meis3* and *hoxa11* mature mRNAs (UTRs not shown). Thin vertical black bar, exon-exon boundary; green box, MEIS domain; blue box, homeodomain; red bar, riboprobe position.
