# Supplementary material for: Expression of meis and hoxa11 in dipnoan and teleost fins provides new insights into the evolution of vertebrate appendages
Source: EvoDevo. 2018 Apr 27;9:11. doi: 10.1186/s13227-018-0099-9 (PMC5924435; doi:10.1186/s13227-018-0099-9)
Supplement: Supplementary file 4 — Additional file 4: Fig. 4 meis3 expression in developing pectoral fins of Neoceratodus. Transversal sections of in situ results at st. 42 and 47. [file 13227_2018_99_MOESM4_ESM.pdf]

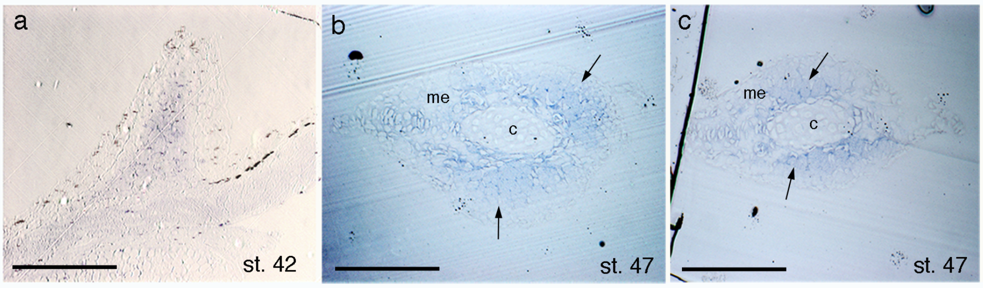

Additional file 4: Figure 4. *meis3* expression in developing pectoral fins of *Neoceratodus*. (a) Cross section through the proximodistal fin bud axis. (b,c) Cross section in a plane perpendicular to the proximodistal axis of the fin depicted in Fig. 2i at (b) proximal and (c) distal level. The black arrows indicate the outer margin of mesenchymal meis3 expression. Abbr.: c, cartilage; me, mesenchyme. Scale bar: 100 µm.
