# Supplementary material for: Expression of meis and hoxa11 in dipnoan and teleost fins provides new insights into the evolution of vertebrate appendages
Source: EvoDevo. 2018 Apr 27;9:11. doi: 10.1186/s13227-018-0099-9 (PMC5924435; doi:10.1186/s13227-018-0099-9)
Supplement: Supplementary file 5 — Additional file 5: Fig. 5 hoxa11 expression in lungfish. In situ results in nervous system, tail and digestive tract. [file 13227_2018_99_MOESM5_ESM.pdf]

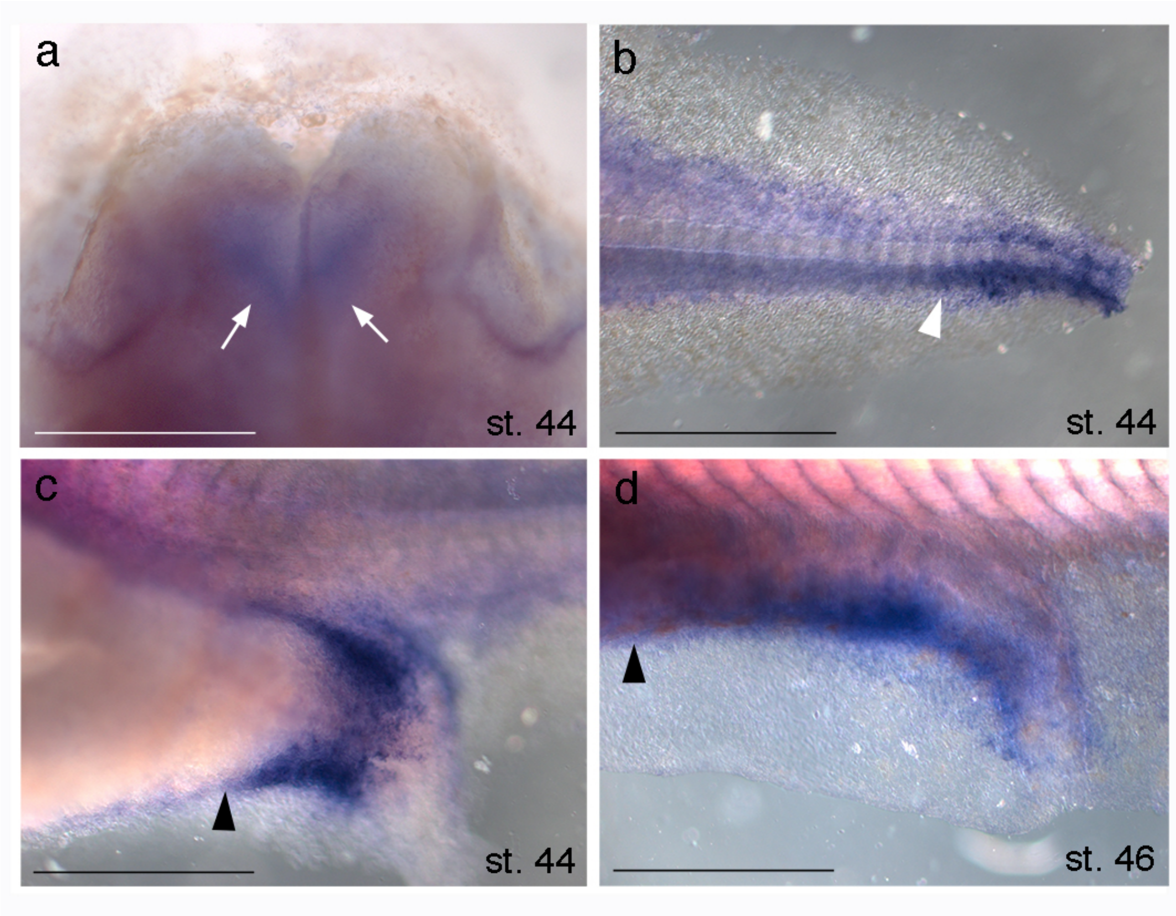

Additional file 5: Figure 5. *hoxa11* expression in lungfish. (a) Frontal view of the neural tube with white arrows showing faint mRNA labelling; dorsal to top. (b) White arrowhead points to expression in posterior somites; dorsal to top, anterior to left. (c,d) Black arrowheads indicate anterior limit of the transcript signal near the distal opening of the digestive tract; dorsal to top, anterior to left. Scale bars: 150 µm.
